# Risk factors and hearing outcomes in infants and young children in KwaZulu-Natal, South Africa

**DOI:** 10.4102/sajcd.v71i1.1031

**Published:** 2024-08-12

**Authors:** Nasim B. Khan, Lavanithum Joseph

**Affiliations:** 1 Discipline of Audiology, School of Health Sciences, University of KwaZulu-Natal, Durban, South Africa

**Keywords:** high risk factors, risk based screening, hearing outcomes, contextual relevance, early hearing detection and intervention, targeted hearing screening

## Abstract

**Background:**

Targeted new-born hearing screening, based on high risk factors is recommended in the absence of universal new-born hearing screening in resource-constrained settings. The relevance of risk factors listed in the guidelines of high-income countries and used by *low-middle income countries* remains relatively unknown. Risk factors consistent with the epidemiological profile, evolution of risks and disease burden in these countries need to be considered.

**Objectives:**

This study aimed to profile the frequency of risk factors and their manifestation in hearing outcomes *of young* children in the KwaZulu-Natal province of South Africa.

**Method:**

A chart review of *N* = 1433 patients’ archival audiology records was conducted, conveniently sampled from a single tertiary hospital (*n* = 351), a provincial assessment and therapy centre (*n* = 649), a university clinic (*n* = 291), and two schools for the deaf (*n* = 142).

**Results:**

Overall, 56% of the participants presented with either a conductive, sensorineural or a mixed hearing loss; 62% of the children had between 1 and 2 risk factors present (Mean [*M*] = 1.1; standard deviation [s.d.] = 0.98). Admission to neonatal intensive care unit, maternal infections, bacterial and viral infections and chemotherapy, from the Joint Committee on Infant Hearing list of high risk factors were significantly associated with hearing loss (*p* < 0.05). Known non-JCIH risks, emerging risks and other statistically significant contextually relevant risk factors were also noted.

**Conclusion:**

Understanding the profile of high risk factors in a given context has implications for *prevention*, early hearing identification and intervention services.

**Contribution:**

Targeted new-born hearing screening needs to be based on risk factors that are contextually relevant. This study is one of the first profiling high risk factors for hearing loss in children in KZN, the province with the second highest population in South Africa.

## Introduction

Hearing loss is one of the most frequently occurring birth defects, affecting approximately 15.5 million children under the age of 5 years worldwide (Shrivastava et al., 2016). It is referred to as the silent, largely discounted epidemic of developing countries given its invisible nature that prevents detection through routine clinical procedures (Petersen & Ramma, 2015; Swanepoel et al., 2009). Almost half of the cases of disabling childhood hearing loss have preventable causes and commonly occur in low- to middle-income countries (LMICs) (Shrivastava et al., 2016). Children in lower socioeconomic contexts have a higher incidence of preventable hearing loss, such as that because of middle ear infections, frequently exacerbated by limited access to healthcare resources (De Voe et al., 2009). This is compounded by the absence of hearing screening programmes, poor resource availability, and the limited awareness of hearing loss and its deleterious effects on children (Galhotra & Sahu, 2019). Additionally, hearing loss attributed to post-natal causes such as infectious diseases are also generally more prevalent in LMICs (Tharpe & Seewald, 2016). Poor prevalence and aetiological data for hearing loss, a lack of an efficient data management system and resource constraints continue to be obstacles to gain support to treat childhood hearing loss and plan for appropriate service delivery (Farr et al., 2017). If a hearing loss is identified late or not managed in time, it poses a risk to crucial quality of life measures such as social participation, educational and vocational attainment (Farr et al., 2017; Michal & Khoza-Shangase, 2014; Olusanya, 2011; Olusanya & Newton, 2007; Olusanya et al., 2004; Swanepoel et al., 2004). Timely management of infants with hearing loss is critical to reducing hearing disability and attaining optimal communication and health outcomes (Friderichs et al., 2012). Breakthrough scientific and technological advances that are quick to administer, consistent and accurate have offered prospects to identify hearing impairments in infants immediately after birth (Gomes & Lichtig, 2005).

Universal new-born hearing screening (UNHS) offered within the first month of life, preferably before hospital discharge, is extensively endorsed in developed countries contexts as a standard of care facilitating early detection and intervention for the majority of infants with permanent childhood hearing loss (PCHL) (Petersen & Ramma, 2015; Swanepoel et al., 2009). The adoption of the same approach in developing regions such as in sub-Saharan Africa, which constitute a disparate burden of PCHL globally, remains unclear (Olusanya, 2011) despite there being documented benefits to early identification (Olusanya, 2011; Olusanya et al., 2004; Swanepoel et al., 2009). Ideally, hearing loss must be identified, diagnosis confirmed and an intervention commenced before the child is 9 months old to capitalise on the critical period for speech and language development (Health Professions Council of South Africa [HPCSA], 2007, 2018; Olusanya, 2012; Olusanya et al., 2004). The Joint Committee on Infant Hearing (JCIH) states that in developed countries, screening must be performed by 1 month of age, diagnosis completed at 3 months, and the intervention should commence by 6 months (Imam et al., 2013; JCIH, 2007).

The JCIH, an organisation consisting of a number of professional associations in paediatric hearing healthcare in the United States of America (USA) has characterised risk indicators often associated with infant and childhood hearing loss. Although their influence is restricted to the USA, its periodic position statements serve as reference documents globally and have been widely used both in developing and developed countries (Olusanya, 2011), representing the international gold standard (Olusanya, 2015). The JCIH promotes using targeted new-born hearing screening (TNHS) where a lack of financial, human and equipment resources limit the development of UNHS (JCIH, 2000, 2007; Olusanya, 2011; Olusanya et al., 2004). The TNHS method seeks to identify and test all infants who are considered at risk of permanent childhood hearing loss, based on established high risk factors (Olusanya et al., 2004). Risk indicators are also suggested for ongoing surveillance of infants as an interim solution where UNHS is not immediately feasible (Olusanya et al., 2005). However, despite the JCIH revising their risk factors on a continuous basis, Korres et al. (2006) argue that it should not be deemed a ‘gold standard’ for all countries, as these might vary greatly. Globally, there is a lack of consensus on the relative importance of the risk factors that have been used to screen infants with hearing loss, and it is therefore important that these are continuously refined for each context (Imam et al., 2013). According to Ganek et al. (2022), the JCIH risk factor lists and/or registers were developed predominantly with high-income countries (HICs) in mind, and therefore risk factors that may be important to LMICs may not be included. Risk factors and risk registries, despite their limitations are still important in identifying infants with possible congenital hearing loss and post-natal loss in the South African context.

In the absence of UNHS, the implementation of risk-based screening, that is, TNHS is a feasible interim and complementary approach to adopt (Kanji, 2019). While TNHS makes intuitive sense, it is created on a set of risk factors not yet confirmed and endorsed in these LMICs (Olusanya, 2011); however, this trend is slowly changing as the need to tailor risk factors to one’s context is being widely recognised (Kanji & Khoza-Shangase, 2019). Based on the advancement of care and treatment over time in developed contexts, risks factors have evolved. Thus, revising the risk factors to reflect current clinical practice is necessitated. For example, care for premature babies in neonatal intensive care units (NICUs) has improved and so has the practice of dispensing ototoxic drugs positively influencing acquired hearing loss. It may thus be claimed that risk factors for hearing loss in developed countries are actually being reduced or eliminated because of advanced medical care and counselling programmes (Biswas et al., 2012). However, risks related to unfavourable birth conditions such as high rates of low birth weight (LBW) and severe hyperbilirubinemia exacerbate risk factors for childhood hearing loss in LMICs and are still vastly relevant (Olusanya, 2015; Olusanya & Okolo, 2006; Tharpe & Seewald, 2016). In addition, vaccine-preventable infections including rubella and meningitis, associated with sensorineural hearing loss in children, occur frequently in these developing contexts (Shrivastava et al., 2016).

Some emerging distinctive risk factors such as undernutrition, maternal high blood pressure and unskilled birth attendants associated with congenital and early onset hearing loss have been reported in Nigeria (Olusanya, 2012; Olusanya & Somefun, 2009). These aspects, combined with a higher incidence of existing risk factors inclusive of birth trauma, asphyxia, neonatal jaundice and ototoxicity are linked to poor maternal and child health services characteristic of many developing contexts (Olusanya, 2011; Olusanya & Somefun, 2009). Because the epidemiological profile of PCHL differs regionally and particularly in developing contexts, profiling the risk factors in young children is an important undertaking (Olusanya, 2011).

Relevant research is required within the South African context to determine high risk factors at play for children with hearing loss, leading to possible modification of existing high risk registers for hearing loss. This will ensure appropriate, timely referrals among the relevant healthcare practitioners within a risk-based hearing screening programme (Kanji & Khoza-Shangase, 2012). Given the unavailability of hearing screening programmes, as well as limited prevalence of information and poor data management in programmes that do exist (Meyer et al., 2012), the risk factor profile of childhood hearing loss in South Africa remains predominantly unknown. With the exception of studies conducted in schools for the deaf in South Africa many decades ago (Sellars & Beighton, 1983, 1976), there is very limited current data related to risk factors for childhood hearing loss available, which is also affected by sample size constraints, or specific patient profiles and contexts limiting generalisability (Le Roux et al., 2015; Kuschke et al., 2020; Storbeck et al., 2023; Swanepoel et al., 2013).

This purpose of this study is to provide one of the first reports on the profile of childhood hearing loss in the KwaZulu-Natal (KZN) province of South Africa by describing the risk factors present in children with confirmed permanent and non-permanent hearing loss. The process of profiling risk factors will lend itself to modification and development of a relevant stratified checklist for screening and monitoring of hearing loss in infants at community level.

### Aim

This study aimed to determine the risk factors and associated hearing outcomes in infants and young children receiving audiology services in KZN, South Africa. Objective one entailed a description of the hearing outcomes or status, and objective two related to describing the risk factors present.

## Research methods and design

### Research design

A descriptive retrospective record review study of archival audiology files of young children attending services from 01 January 2012 to 31 December 2016 (5-year period) was conducted. Audiology records included audiograms and audiologist’s notes. Data for the audiological test battery included available otoscopy, tympanometry, pure tone and speech audiometry, behavioural audiometry, auditory brainstem response (ABR), auditory steady state response (ASSR), and otoacoustic emission (OAE) results.

### Sampling

The retrospective record review was conducted in the audiology departments of a tertiary hospital (uMgungundlovu District), a provincial assessment and therapy centre (eThekwini District), a university clinic (eThekwini District), and two schools for the deaf. One school was in the eThekwini District and the other in the Ugu District of KZN. The sites were conveniently sampled to enable the results to be representative of the province’s demographics, and to enable their generalisability to the KZN population. The study included infants and children up to 7 years of age who attended the health facilities for a hearing test, irrespective of ethnic or language group. The cut-off age and grade for those attending schools was grade four, and up to 12 years of age because of late diagnosis and intervention.

Relevant records of audiologists and other clinicians involved in the management of the child were perused for information related to biographical and case history information, risk factors, age of suspicion, identification, diagnosis and management; as well as type and severity of loss. Files with more than 10% of the above data categories that were not recorded, as well as files of young children only receiving screening with no follow up diagnostic and management services were excluded. The Gelfand (2009) classification of degree of hearing loss was used, taking into account the pure tone average of the better ear (Pure-tone average < 15 dB normal hearing; 16 dB – 25 dB slight hearing loss; 26 dB– 40 dB mild hearing loss; 41 dB – 55 dB moderate hearing loss; 56 dB – 70 dB moderately severe hearing loss; 71 dB – 90 dB severe hearing loss and ≥ 90 dB profound hearing loss). A statistician, Dr Ben Sartorius, was consulted to determine the sample size for the study.

It was determined that for 80% power and 95% confidence level, one needed to randomly sample 1240 records. The final number reviewed was 1433 records, a sample larger than the number of records for a statistically sound study. For the hospital, assessment and therapy centre and the university clinic, the audiology clinic diaries were perused and every second patient file that met the inclusion criteria was randomly chosen, until the desired number of records were obtained. For the schools, all records of children in the current preschool classes up to Grade 4 were included. These files were selected randomly using a lottery method whereby files were pulled out of a box until the desired number of files were reached.

### Description of the participants per record

At the tertiary hospital 480 records were reviewed (accessed files from 3 years of diary entries 2014–2016) of which 351 files were included. A total of 1031 files were reviewed at the assessment and therapy centre and 649 files were included (accessed files from the past 5 years of diary entries, 2012–2016). At the university clinic, 400 files were reviewed of which 291 files were included (accessed files from 4 years of diary entries 2013–2016). From the schools 168 records were reviewed but only 142 files were included.

The majority of reviewed files, 649 (45%) were from the assessment and therapy centre followed by 351 files (24%) obtained from the tertiary hospital. Most of the referrals received at the institutions (*n* = 871, 81.5%) were from other health facilities. Of the files sampled, 55.5% (*n* = 796) were of males. The age range most commonly sampled was 3 years and younger with 706 files (49 %). The majority of the files reviewed (*n* = 1163, 81.2%), indicated data were of isiZulu speakers. Furthermore, 1096 (98.6%) of the sample were classified as poor and indigent, with a H_0_ classification as per the Department of Health’s classification system. See Table 1 for more information related to the demographic and case history information recorded in the files. All categories of information had different *n* values, as these may not have been recorded in every file. For example, birth weight information was only available in 911 records. Any ear-related signs and symptoms that were recorded in the files were also observed and included as part of the case history information.

**TABLE 1:** Summary of biographical information.

Demographic and case history information	*n*	%
**Data Sources (*N* = 1433)**		
Assessment centre KZN-DOH	649	45.0
Tertiary public hospital	351	24.0
University clinic	291	20.0
Two schools for the deaf	142	11.0
**Gender (*N* = 1433)**		
Male	796	55.5
Female	637	44.5
**Age categories (years) (*N* = 1443)**		
< 2	333	23.0
2–3	373	26.0
3–5	306	21.0
5–7	339	24.0
> 7	82	6.0
**Spoken language (*N* = 1433)**		
isiZulu	1163	81.2
English	256	18.0
Other	14	1.0
**Income classification by DOH (*N* = 1107)**		
H_0_	1096	99.0
H1	1	0.1
H2	9	0.8
H3	1	0.1
**Sources of referrals (*N* = 1072)**		
Other public health institutions	871	81.5
Self-referrals	133	12.5
Teachers	35	3.0
Other healthcare practitioners	33	3.0
**Birth weight (kg) (*N* = 911)**		
Below 1.5	328	36.0
Above 1.5	583	64.0
**Apgar score at 5 min (*N* = 425)**		
0–6	142	33.0
7–10	283	67.0
**Type of delivery (*N* = 1056)**		
Full term	473	44.8
Premature	577	54.6
Overdue	6	0.6
**Method of delivery (*N* = 679)**		
Normal vaginal delivery	455	67.0
Caesarean section delivery	215	32.0
Breech delivery	9	1.3
**Ear-related signs and symptoms (*N* = 54)**		
Ear pain	24	44.0
Itchy ears	9	16.0
High fever	21	40.0

*Abbreviations*: DOH, Department of Health; KZN, KwaZulu-Natal.

### Data collection method, procedure and analysis

The researcher used an electronic data sheet for the audiology record review. The data sheet was developed using elements from studies by Olusanya et al. (2008), Rout and Singh (2010), Swanepoel et al. (2013), the JCIH (2007) position statement, and the Early Hearing Detection and Intervention (EHDI) position statement (HPCSA, 2007). The version of both the HPCA EHDI position statement and the JCIH position statement, available at the time of data collection, were used and referenced as such, instead of the later versions currently available. The following sections were included: biographical and case history information, risk factors that caused or contributed to the hearing loss and the type and degree of the hearing loss after diagnosis.

Permission to conduct the retrospective audiology record review for the pilot study and main study at the health facilities was obtained from the Department of Health and Hospital Managers and for the University Audiology Clinic from the University Registrar and Head of Department for Audiology. Permission to access the school records was obtained from the Provincial Department of Education and the School Principals. The researcher conducted the data collection process, in approximately 40 days, over a period of 18 months from June 2016 to November 2017.

A pilot study was conducted prior to the main study. For the retrospective review, 12 audiology records were reviewed at the university clinic, the assessment and therapy centre, the tertiary hospital and the schools. This enabled the researcher to identify the items routinely collected at the institutions to be checked against the data sheet developed for the study, the usefulness, as well as the user-friendliness of the data sheet. These results, however, were not included in the main study. The results further indicated that each site recorded slightly different data in their files and therefore the researcher decided to retain all the data elements originally envisaged for the study and did not make any changes to the data sheet. Because of the comprehensive nature of the data sheet, no new elements were added following the pilot study.

In addition, approximately 10% of the files were rechecked and re-recorded by the researcher to check if the data captured from the same file on a previous occasion were the same, for reliability purposes. Every fifth entry of 50 entries was chosen for a recheck. The kappa statistic was used to test interrater reliability (McHugh, 2012). The overall kappa score was between 0.81 and 1 showing a high agreement between the sets of data entries for 140 sets for records that were rechecked.

Data were analysed using descriptive statistics including frequency and/or percentage counts and frequency distribution tables. The means, standard deviations (s.d.) and the maximum and minimum scores were documented for the risk factors. The Chi-square (χ^2^) test was used to identify any significant association between categorical explanatory (risk factor) variables and hearing loss. Multivariable logistic regression could not be employed to adjust for the influence of multiple explanatory and/or confounding variables, because of missing data related to risk factors.

### Ethical considerations

Approval from the University of KwaZulu-Natal (UKZN) Biomedical Research Ethics Committee (BREC: REF: BE 395/14) was obtained to conduct this research study. Following this, permission from the relevant gatekeepers was obtained. The researcher provided information regarding the nature of the study to the sites. The researcher developed a research coding system in order to ensure that the identity of the participants remained protected and/or confidential. Names and file numbers are not disclosed. The electronic data sheets are kept electronically and are password protected. The data extraction sheets are kept in a locked cabinet within the Discipline of Audiology at UKZN and will only be accessed by the researcher of the project and the supervisor for the purposes of the study. The data extraction sheets will be kept for a period of 5 years and will thereafter be destroyed.

## Results

The results of the study are presented according to the two objectives: (1) a description of the hearing outcomes and (2) the description of risk factors present.

### Objective 1: Description of hearing outcomes

Hearing status, unconfirmed losses and those not recorded in file (NRIF) are summarised in Table 2. Losses were classified according to the types of hearing loss, namely conductive hearing loss (CHL), sensorineural hearing loss (SNHL), and mixed hearing loss (MHL). This was further classified according to the data for the unilateral right and left ears, respectively, and bilaterally. In the right ear, 32.4% (*n* = 464) had normal hearing, 55.8% (*n* = 799) had some type of hearing loss and the remainder was either not confirmed or had missing information. In the left ear, 31.8% (*n* = 455) had normal hearing, with 56.4% (*n* = 808) having some form of hearing loss.

**TABLE 2:** Overall hearing status (*N* = 1433).

Hearing status	Unilateral right ear (*N* = 1433)		Unilateral left ear (*N* = 1433)		Bilateral (*N* = 1416)	
	*n*	%	*n*	%	*n*	%
Conductive HL	409	28.5	401	28.0	401	28.0
Sensorineural HL	288	20.1	298	20.8	288	20.1
Mixed HL	102	7.1	109	7.6	102	7.1
Normal hearing	464	32.4	455	31.8	455	31.8
Not confirmed	159	11.1	159	11.1	159	11.1
NRIF	11	0.8	11	0.8	11	0.8

*Abbreviations*: HL, hearing loss; NRIF, not recorded in file.

Of the 799 participants who had a hearing loss in the right ear, the majority 51% (*n* = 409) was because of CHL. In the left ear of the 808 participants who had a hearing loss similarly, 50% (*n* = 401) was because of CHL.

With regard to the severity of hearing loss for the right ear unilateral hearing loss (*N* = 799), 30% (*n* = 237) was slight loss followed by 17% (*n* = 136) which was a mild loss. Severe and profound losses combined made up about a third (32%) of the participants. For the left ear unilateral hearing loss (*N* = 808), 29% (*n* = 236) had a slight hearing loss followed by 17% (*n* = 135) with a mild hearing loss. The combined severe and profound losses was 34% (*n* = 168) (see Table 3).

**TABLE 3:** Severity of hearing loss (*N* = 1433).

Hearing loss	Intensity	Unilateral right ear (*n* = 799)		Unilateral left ear (*n* = 808)		Total right and left ears (*N* = 1607)	
		*n*	%	*n*	%	*n*	%
Slight	16 dB – 25 dB	237	30.00	236	29.00	473	29.00
Mild	26 dB – 40 dB	136	17.00	135	17.00	271	19.00
Moderate	41 dB – 55 dB	110	14.00	106	13.00	216	13.00
Moderately severe	56 dB – 70 dB	62	0.08	63	0.08	125	0.08
Severe	71 dB – 90 dB	135	17.00	134	17.00	269	17.00
Profound	≥ 91 dB	119	15.00	134	17.00	263	16.00

### Objective 2: Description of the risk factors

The second objective provides a description of the risk factors present in the files reviewed, risk count and statistically significant risk factors per type of loss.

#### Risk factors prevalent

The risk factors included the JCIH (2007) list of risk factors, known non-JCIH risk factors (Olusanya, 2011), emerging risk factors (Olusanya, 2011), and South African specific risk factors (HPCSA, 2007) (see Table 4). Other medical risk factors that may or may not be related to hearing loss were also documented. In the majority of the files, the information related to risk factors were not always captured. Thus, it is unclear if this was because of the child not presenting with the risk factor, if the risk factors were unknown or if the audiologist did not obtain this information.

**TABLE 4:** Risk factors associated with hearing loss found in records (*N* = 1433).

Risks	*n*	%
**JCIH risk factors** †		
Caregiver concern regarding hearing, speech, language, or development delay	427	30.0
Family history of permanent childhood hearing loss	95	7.0
Child in neonatal intensive care for greater than 5 days	483	34.0
Exposure to ototoxic medication	405	28.0
Hyperbilirubinemia requiring exchange transfusion	133	9.0
Maternal infections	54	4.0
Craniofacial anomalies	122	8.5
Syndrome known to include a hearing loss	90	6.0
Neurodegenerative disorder	38	3.0
Bacterial or viral meningitis	99	7.0
Head trauma, requiring hospitalisation	3	0.0
Chemotherapy	73	5.0
Mechanical ventilation	39	3.0
**Known non-JCIH risk factors‡**		
Consanguineous marriage	2	0.13
**Emerging risk factors‡**		
Mother had hypertensive disorders in pregnancy	135	9.0
**South Africa specific risk factors§**		
Recurrent otitis media	620	43.0
HIV infected or affected	233	16.0
**Other general medical risk factors**		
Prematurity	577	40.2
Low birth weight	328	22.7
Unspecified exposure to viral disease	296	21.0
Mother took medication during pregnancy (ARV, TB)	266	18.5
C-section deliveries	215	15.0
Child had other infection not specified	208	14.5
Asphyxia (encephalopathy)	198	13.8
Apgar score (0–6) at 5 min	142	10.0
Klebsiella sepsis	126	8.0
Frequent flus and colds	94	6.5
Mother suffered trauma during pregnancy	87	6.0
Twin pregnancy	67	4.6
Preeclampsia	21	1.4
Hydrocephalus	20	1.3
Placenta praevia	11	0.07
RH incompatibility	11	0.07

*Abbreviations*: ARV, antiretroviral; HIV, human immunodeficiency virus; HPCSA, Health Professions Council of South Africa; JCIH, Joint Committee on Infant Hearing; RH, rhesus; SA, South Africa; TB, tuberculosis.

† , risk factors as indicated by Joint Committee on Infant Hearing. (2007). Year 2007 position statement: Principles and guidelines for early hearing detection and intervention programs. *Pediatrics, 120*(4), 898–921. https://doi.org/10.1542/peds.2007-2333

‡ , risk factors as indicated by Olusanya, B.O. (2011). Making targeted screening for infant hearing loss an effective option in less developed countries. *International Journal of Otorhinolaryngology, 75*(3), 316–321. https://doi.org/10.1016/j.ijporl.2010.12.002

§ , risk factors as indicated by Health Professions Council of South Africa (HPCSA). (2007). *Early Hearing Detection and Intervention (EHDI) guidelines 2007*. Retrieved from https://www.hpcsa.co.za/Early_Hearing_Detection_and_Intervention_(EHDI)

The most frequently occurring risk factors from the JCIH (2007) list included the child being in NICU for over 5 days, caregiver concern related to delayed speech and language development, exposure to ototoxic medication, hyperbilirubinemia requiring exchange transfusion, craniofacial anomalies, bacterial and viral infections, and family history of hearing loss. For the known non-JCIH risk factors, emerging risks and South African specific risks, it was recurrent otitis media, human immunodeficiency virus (HIV) infected and affected, and maternal hypertension. The most frequent risk factors from the other medical risks recorded, included prematurity, LBW, unspecified exposure to a viral disease, and the mother taking ototoxic medication during pregnancy. Some of these general medical risk factors could co-occur with some of the risk factors for hearing loss and increase the likelihood of a hearing loss and were therefore included.

There were 90 children in this sample with a syndromic profile and this was analysed further. Most of these children (*n* = 65) presented with Down’s syndrome (72%), followed by Treacher Collins syndrome and other syndromes as summarised in Table 5 (Section A). Some of the developmental conditions found in the records included epilepsy, cerebral palsy, cleft lip and palate, and other syndromes not directly related to hearing loss, which are also summarised in Table 5 (Section B).

**TABLE 5:** Syndromes associated with hearing loss (*N* = 90) and other developmental conditions (*N* = 212).

Syndromes and developmental conditions	*n*	%
**Section A: Syndromes (*N* = 90)**		
Waardenburg syndrome	5	6.0
Dandy–Walker syndrome	4	4.0
Pierre Robin syndrome	1	1.0
Treacher Collins syndrome	6	7.0
Goldenhar syndrome	1	1.0
CHARGE syndrome	3	3.0
Branchio-oto-renal syndrome	1	1.0
Brachman de Lange syndrome	1	1.0
Mosaic syndrome	1	1.0
Noonan’s syndrome	1	1.0
Roberts syndrome	1	1.0
Usher syndrome	1	1.0
Downs syndrome	65	72.0
**Section B: Developmental conditions (*N* = 212)**		
Epilepsy	129	61.0
Cerebral palsy	35	17.0
Cleft lip and palate	28	13.0
Other syndromes not related to hearing loss	20	9.0

*Abbreviation*: CHARGE, coloboma, heart defect, atresia choanae, restricted growth and development, genital abnormality, and ear abnormality.

Maternal infections (*n* = 54) were also analysed further. The most common of the maternal infections prevalent in this sample was cytomegalovirus (CMV) with 54% (*n* = 29) followed by maternal rubella with 26% (*n* = 14) as summarised in Table 6.

**TABLE 6:** Maternal infections present in the current cohort (*N* = 54).

Maternal infections	*n*	%
Cytomegalovirus (CMV)	29	54.0
Rubella	14	26.0
Syphilis	5	9.0
Toxoplasmosis	4	7.0
Herpes	2	4.0

#### Risk count

Twenty-seven per cent of the children had no JCIH (2007) risk factors present. Most children (*n* = 643) had at least one of the 11 JCIH (2007) risk factors (45%), relating to either a permanent congenital hearing loss or a late-onset progressive hearing loss present. This was followed by 19% of the children with two risks of the JCIH (2007) list of risk factors. The total counts of JCIH (2007) risk factors prevalent in this sample is captured in Table 7. The range for the presence of JCIH (2007) risk factors were between zero and five. The mean was 1.12, with a s.d. of 0.9, and 95% confidence interval (CI; 1.07, 1.17).

**TABLE 7:** Total counts of Joint Committee on Infant Hearing (JCIH) risk factors (*N* = 1433).

Number of JCIH risk factors†	*n*	%
1	643	44.9
2	277	19.3
3	86	6.0
4	28	2.0
5	10	0.7

† risk factors as indicated in Joint Committee on Infant Hearing. (2007). Year 2007 position statement: Principles and guidelines for early hearing detection and intervention programs. *Pediatrics, 120*(4), 898–921. https://doi.org/10.1542/peds.2007-2333

#### Significant risks per type of loss as determined with Chi squared associations and odds ratio

Chi squared associations were calculated and for those who showed a significant association, the odds ratios were generated. These were performed for the JCIH 2007 list and all other risk factors as per the type of hearing loss as depicted in Table 8. For the JCIH risk factors (Table 8, section A), NICU admission was significant for both CHL and SNHL. Maternal infection, bacterial infection and chemotherapy were significantly associated with SNHL and jaundice for MHL.

**TABLE 8:** Joint Committee on Infant Hearing† and all other risk factors per type of loss – Odds ratios and statistically significant risks only.

Risk factors	Test of association (Χ^2^)	Odd ratio	95% CI	*p*
**Section A: JCIH risk factors**				
**CHL**				
NICU admission > 5 days	0.036	2.011	1.037–3.902	0.039
**SNHL**				
NICU admission > 5 days	0.037	0.499	0.351–0.710	0.000
Maternal infection	0.000	4.236	1.851–9.695	0.001
Bacterial infection	0.046	8.123	4.029–16.376	0.000
Chemotherapy	0.000	6.900	2.981–15.968	0.000
**MHL**				
Maternal infection	0.000	5.644	2.172–14.664	0.000
**Section B: All other risk factors**				
**CHL**				
Birth weight	0.002	2.060	1.497–2.834	0.000
Mother on ototoxic drugs	0.017	3.149	2.152–4.608	0.000
Recurrent ear infections	0.000	46.176	30.781–69.271	0.000
HIV exposure	0.000	8.112	4.100–16.050	0.000
Cleft lip/palate	0.002	4.226	1.555–11.487	0.005
**SNHL**				
HIV exposure	0.000	5.962	2.802–12.683	0.000
Low Apgar	0.000	7.311	4.301–12.426	0.000
Prematurity	0.000	1.908	1.580–2.304	0.000
Method of delivery	0.000	1.414	1.150–1.740	0.001
**MHL**				
Recurrent OM	0.000	23.876	13.743–41.482	0.000
HIV exposure	0.000	17.178	8.012–36.831	0.000
Low Apgar	0.000	6.413	3.223–12.762	0.000
Ototoxic medication	0.000	12.659	5.138–31.189	0.000

*Abbreviations*: CHL, conductive hearing loss; CI, confidence interval; HIV, human immunodeficiency virus; JCIH, Joint Committee on Infant Hearing; MHL, mixed hearing loss; NICU, neonatal intensive care unit; OM, otitis media; SNHL, sensorineural hearing loss.

† risk factors as indicated in Joint Committee on Infant Hearing. (2007). Year 2007 position statement: Principles and guidelines for early hearing detection and intervention programs. *Pediatrics, 120*(4), 898–921. https://doi.org/10.1542/peds.2007-2333

For the JCIH risk factors, for example, a child who had NICU admission over 5 days was 2.011 times more likely to have a CHL. This was statistically significant (*p* = 0.039). A child who had maternal infection was 4.236 times more likely to have a SNHL, for bacterial infection 8.123 more likely to have a SNHL, and for children undergoing chemotherapy 6.900 times more likely to have a SNHL. The overall odds were statistically significant for all (*p* < 0.001). For all other risk factors including known non-JCIH risk factors (Table 8, section B) for CHL, birth weight, mother on ototoxic medication, HIV exposure, recurrent ear infections and cleft palate were significant. For SNHL, a low Apgar score and prematurity were also significant. For MHL, recurrent otitis media and exposure to ototoxic medication were significant in addition to HIV and low Apgar scores.

## Discussion

In this study, of the JCIH (2007) risk factors, the child being in NICU over 5 days, caregiver concern regarding delayed speech and language development, exposure to ototoxic medication, jaundice, craniofacial anomalies, bacterial and viral infections as well as family history featured as some of the risk factors most frequently mentioned. Another study conducted with five cochlear implant programmes in South Africa, with 264 paediatric cochlear implant recipients, found that the most prevalent risks were NICU admission (28.1%), followed by family history and prematurity (Le Roux et al., 2015). These findings are similar to this study. A statistically significant relationship between NICU admission for both CHL and SNHL was observed in this study.

A study conducted in the Gauteng province found that the most common risk for SNHL was family history, admission to NICU for longer than 5 days, asphyxia and jaundice (Swanepoel et al., 2013). Hyperbilirubinemia was also a common post-natal risk factor with 2.3% of children requiring blood transfusion in the study performed by Le Roux et al. (2015). When blood transfusion is necessary, excessive waste products from the liver end up in the blood, eventually manifesting in high bilirubin levels (hyperbilirubinemia), which being ototoxic may result in a hearing loss (JCIH, 2019). Hyperbilirubinemia was closely followed by meningitis as a risk factor that occurred in 10% of the children in the Le Roux et al. (2015) study.

These two risk factors also featured in the top seven JCIH (2007) list of risk factors that occurred most frequently in this study. A study carried out in Kenya by Karanja et al. (2013) showed a high prevalence of SNHL in children treated for bacterial meningitis, 36 out of 83 participants (44.4%) had some form of hearing loss with 14 (16.9%) having a severe to profound loss. The dire need for timely identification and diagnosis for children at a high risk of acquired hearing loss because of illnesses such as meningitis is unique to the African context (Moodley & Storbeck, 2015). This risk factor was also found to be statistically significant for SNHL in this study. One of the significant factors is the age at which the hearing loss occurs as this affects rehabilitation needs. The younger the child, the greater the impact on speech and language development (Karanja et al., 2013).

In this study, just over 50% had CHL, just over one third (36%) had SNHL, and 13% had MHL. A study by Kushke et al. (2020) conducted in the Western Cape Province similarly found that the majority of children also presented with CHL (64.6%), followed by SNHL (28.7%) – slightly lower than in this study. Overall, the most common risk factor for CHL in this study was otitis media, which concurs with the study conducted by Kuschke et al. (2020). A study conducted by Biagio et al. (2014) at a primary healthcare clinic in Gauteng province, found a prevalence of chronic suppurative otitis media (CSOM) to be high at 6.6%. Because of the adverse effects that living conditions and environmental risks have on health and the increased risk this poses for possible hearing loss, it is important for children to be screened for hearing loss in LMICs. A pilot study investigating hearing screening outcomes in a group of paediatric patients attending an HIV/AIDS clinic at a hospital in Gauteng also found otitis media to be the most prevalent cause of hearing loss in these HIV infected patients (Khoza-Shangase & Turnbull, 2010). Both conditions present a major burden to ear and hearing care services in South Africa (Sebothoma & Khoza-Shangase, 2020). Conductive hearing loss is also prevalent in other developmental conditions such as cleft lip and palate (Cheong et al., 2016): a relationship found to be statistically significant in this study also.

A major concern with otitis media is not only that it leads to a CHL, but if left untreated or mismanaged this can eventually result in a SNHL (Singh et al., 2003). Maternal and/or infant HIV infection can therefore present a risk for congenital or acquired hearing loss and forms part of the list of risk indicators in South Africa for risk-based screening and surveillance (HPCSA, 2007). Geographical differences in otitis media incidence and prevalence is complex, ranging from numerous host-related factors such as age, gender, race, genetic factors, nutritional and immunity status, as well as environmental factors such as recurrent respiratory tract infections, seasonal variations and exposure to tobacco smoke (Biagio et al., 2014). Furthermore, the prevalence of otitis media associated with low socio-economic conditions is a reality for many children in Africa (Choffor-Nchinda et al., 2020), necessitating hearing screening initiatives.

The JCIH (2007) Position Statement excludes otitis media from its list. However, it recommends careful assessment of middle--ear status at all immunisation visits, and referral of children with persistent middle--ear effusion lasting 3 months or longer. Frequent episodes of otitis media with effusion (OME) resulting in CHL, influences speech and language development negatively and should therefore be monitored (HPCSA, 2018). While the direct effect of HIV exposure in utero on new-born and infant hearing has not yet been established, given its high prevalence in South Africa (HPCSA, 2018) it needs further investigation. A child born to a mother that has HIV/AIDS is at an increased risk for hearing loss for a variety of reasons such as very low birth weight (VLBW), heightened vulnerability for acquiring infections such as meningitis, viral encephalitis and cytomegalovirus (Spiegel & Bonwit, 2002). Sensorineural hearing loss and CHL may also be caused directly by a viral infection (Cohen et al., 2014).

Risk factors such as admission to NICU for over 5 days, asphyxia and extremely low birth weight (ELBW) have also been found to be prevalent in other South African studies. It is interesting to notice, as highlighted in the study conducted by Kanji and Khoza-Shangase (2012), that some of the reasons for the referrals made (e.g., prematurity and birth asphyxia) does not form part of the JCIH (2007) list of risk factors for hearing loss. However, birth asphyxia does feature in the recent JCIH (2019) position statement. Birth asphyxia results when an infant is unable to ‘initiate and maintain spontaneous respiration with subsequent acidosis and hypoxic-ischemic injury to tissues’ (Ogunlesi et al., 2013, p. 31). A low Apgar score is used as a proxy for birth asphyxia in LMICs (Alhazmi, 2023; Olusanya, 2009). An additional risk factor, as identified by Kanji and Khoza-Shangase (2012), included prematurity and was found to be present in 17.5% of their study sample, lower than the 40.0% found in this study. Kanji and Khoza-Shangase (2012) stated that preterm infants with jaundice have a higher risk of developing hearing impairment, even with lower bilirubin levels. Thus, the need for TNHS is highlighted for infants with jaundice and prematurity, because of its combined and synergistic effect on each other. Prematurity alone may not have a severe impact on hearing. However, it is commonly associated with multiple other risk factors (exposure to mechanical ventilation, ototoxic drugs, hypoxia and NICU admission) that can influence hearing. Therefore, the risk of hearing loss in this population is considerably higher than in the general new-born population (Cristobal & Oghalai, 2008). If factors contributing to LBW and prematurity are effectively managed, the possibility of hearing loss can be decreased.

In this study, just over a third of babies (36%) who had birth weight recorded fell below 1500 g. There is an increasing risk in VLBW and ELBW babies because of other co-occurring risk factors (Newton, 2001). Cristobal and Oghalai (2008) and (Newton, 2001) support that VLBW by itself may not adversely affect hearing; however, it commonly co-exists with other risk factors that can alter hearing in a synergistic manner, which includes ototoxic drugs, hypoxia and hyperbilirubinemia. This may lead to early or delayed-onset of SNHL, as well as progression of a mild pre-existing SNHL years after hospital discharge. Engdahl and Eskild (2007) assessed the impact of LBW on the risk of SNHL among children born in Norway and found that LBW of less than 1500 g was linked with SNHL and that the risk decreased with increasing birth weight, illustrating the need for timely audiological evaluations. Similarly and in agreement with the studies above, a recent study on high-risk infants in two Gauteng hospitals based on case history factors found that preterm birth (95.7%) followed by exposure to ototoxic medication (87.7%), neonatal jaundice (80.6%) and birth weight below 1500 g (66.7%) were most prevalent (Kanji & Khoza-Shangase, 2019).

Earlier studies conducted a few decades ago (1976, 1983) assessed 3064 children at schools for the deaf in South Africa and in addition to congenital hearing losses found 25% acquired hearing loss (Sellars et al., 1976; Sellars & Brighton, 1983). The key risks were maternal rubella, jaundice, meningoencephalitis, severe illness, birth trauma and prematurity. About 7% had syndromes, of which 11% were familial risks and 57% were from unknown causes (11% with other anomalies and 46% without other anomalies) (Sellars et al., 1976; Sellars & Brighton, 1983). Common syndromes such as Waardenburg syndrome, Treacher Collins syndrome, Pendred syndrome, Usher syndrome and Branchial Arch syndrome were evident. In this study cohort, most children presented with Down syndrome followed by Treacher Collins syndrome and Waardenburg syndrome. The study by Le Roux et al. (2015) indicated that 1 out of 10 children presented with a syndrome with the most common one being Waardenburg syndrome (comprising about 52% of the cases). In the study by Swanepoel et al. (2013), common syndromes found were Goldenhar syndrome, Cri-du-Chat syndrome and Prader Willi syndrome. A high prevalence (21, 4%) of auditory neuropathy spectrum disorder (ANSD) was also found. In Nigeria, ANSD was found to be as high as 16% for children born outside a hospital versus 10% in children born at a hospital (Swanepoel et al., 2013). Furthermore, ANSD was associated with jaundice, asphyxia and LBW. Although there was no mention of ANSD in any of the records reviewed for this study, there were many children who presented with jaundice, asphyxia and LBW, which are associated maternal and child factors, predisposing ANSD (Swanepoel et al., 2013). A study performed in Belgium found a prevalence of 19% ANSD with confirmed hearing loss on ABR testing (Maris et al., 2011). However, in a study conducted in Brazil, 1.2% of ANSD was seen in individuals with SNHL (Penido & Isaac, 2013). Auditory neuropathy spectrum disorder is a challenging hearing disorder with many causal factors that need to be taken into account, necessitating more research in the area.

There are other emerging risk factors from African countries such as Nigeria that need to be investigated further as this may have implications in South Africa also. The most common cause of hearing loss in a Nigerian teaching hospital were attributed to labour complications such as prolonged labour, pre-eclampsia and infections during and after labour (Olusanya, 2011). A study by Bakhshaee et al. (2008) found that preeclampsia may have a temporary effect on hearing in new-borns but its effects on the inner ear needs further investigation. A South African study also found that babies born to mothers reporting hypertension during pregnancy were more likely to have hearing impairment than those who did not (Ramma & Sebothoma, 2016). According to Olusanya (2011), some clinical illnesses require diagnostic protocols such as birth asphyxia, neonatal sepsis and jaundice, rendering it complicated to detect in resource constrained settings. While a low Apgar score is used as a proxy for birth asphyxia in LMICs, these have limitations (Olusanya, 2009). Other clinical decisions, for example that of a diagnosis for neonatal sepsis, are made on clinical judgement that may also be problematic, leading to over or under-referrals when conducting risk-based screening (Olusanya, 2011).

The high prevalence of hearing loss in infants and its deleterious effects on speech and language development render it a major public health concern, necessitating the development of population based new-born screening programmes. In some LMICs, this may be currently unattainable, thus the benefits of early detection and early intervention is lost, predisposing children to poor and negative outcomes. Hence, there needs to be reliance on risk-based screening, which is also important for late-onset and progressive hearing loss, provided they are specific to a given context, including those of LMICs.

## Conclusion

Understanding and awareness of high risk factors for childhood hearing loss, as it pertains to the South African context has repercussions for medical intervention and for the field of audiology as planning for EHDI services for hearing impairment is vital. The JCIH (2007) list of risk factors while applicable in most contexts, is applicable mainly to HICs. Context-specific risk factor identification is important to direct appropriate prevention and intervention strategies. Improved prenatal and postnatal care could reduce NICU stay and jaundice, direct genetic counselling efforts, facilitate educational programmes for mothers and reduce preventable causes of hearing loss (Ganek et al., 2022). Poor and limited services and data management in South Africa is largely unknown along with the associated risk profiles, thus, further research is warranted. Further risk-based studies can be conducted prospectively to gather appropriate data for research goals. An anticipated limitation of retrospective reviews is that because the data are not collected at the time for research, some missing information is likely. Another significant challenge in this study was the lack of reporting on risk factors and the variable manner in which reporting occurred in the files.

Some files had incomplete information as most of the mothers had passed on after the child was born and the current caregivers did not have any information about the pregnancy and birth information affecting the information obtained. The missing data also affected the data analysis. A risk model using logistic regression was not possible as initially envisaged because of the missing data. As data recording was paper-based, the writing was not always legible and this could have affected some of the results. This variability and inconsistent reporting further influences obtaining consensus on the most important risk factors in any given context (Ganek et al., 2022). It is recommended that audiologists routinely take detailed case history information that includes risk factors present for screening, monitoring and surveillance purposes. Risk factors should be continuously updated given the healthcare resources, disease burden and emerging infections. In the absence of UNHS, TNHS based on contextually relevant risk factors needs to be considered in audiological services for young children.
